# Integrated bioinformatics and experiments reveal the roles and driving forces for HSF1 in colorectal cancer

**DOI:** 10.1080/21655979.2021.2018235

**Published:** 2022-01-10

**Authors:** Xiaomin Ren, Liyuan Zhang, Xiaolin Ma, Jiaqiu Li, Zhong Lu

**Affiliations:** a Department of Oncology, Affiliated Hospital of Weifang Medical University, Weifang, China; b Jinming Yu Academician Workstation of Oncology, Clinical Research Center, Affiliated Hospital of Weifang Medical University, Weifang, China; c Department of Clinical Medicine, Medical College of Qingdao Binhai University, Qingdao, China

**Keywords:** HSF1, biomarker, bioinformatics, immune therapy, super-enhancer, HuR

## Abstract

Heat shock factor 1 (HSF1) has watershed significance in different tumors. However, the roles and driving forces for HSF1 in colorectal cancer (CRC) are poorly understood. Our study integrally analyzed the roles and driving forces for HSF1 in CRC by bioinformatics and experiments. The expression and prognostic characteristics of HSF1 were analyzed via UALCAN, GEPIA2, TISIDB, Prognoscan and HPA databases. Then, we analyzed the correlation between HSF1 expression and immune features via TIMER2 database. Subsequently, we explored the driving forces for HSF1 abnormal expression in CRC by bioinformatics and experiments. Our results showed that HSF1 was overexpressed and correlated with poor prognosis in CRC. And the expression of HSF1 was significantly correlated with multiple immune cell infiltration and was negatively correlated with immunomodulators such as programmed cell death 1 ligand 1(PD-L1). Along with many driver genes in particular *TP53*, super-enhancer, miRNA and DNA methylation were all responsible for HSF1 overexpression in CRC. Moreover, we demonstrated that β-catenin could promote the translation process of HSF1 mRNA by interacting with HuR, which could directly bind to the coding sequence (CDS) region of HSF1 mRNA. Collectively, HSF1 may be useful as a diagnostic and prognostic biomarker for CRC. HSF1 was closely correlated with immune features. Genetic and epigenetic alterations contributed to HSF1 overexpression in CRC. More importantly, we demonstrated that HSF1 may be regulated at the level of mRNA translation by β-catenin-induced HuR activity.

## Introduction

Based on the latest data published by the International Agency for Research on Cancer (IARC), about 1.93 million new colorectal carcinoma (CRC) cases and 0.93 million deaths occurred in 2020 worldwide, accounting for about 10% of all new cancer cases and 9.4% of cancer deaths [1]. Furthermore, the morbidity and mortality of CRC is still rising in many countries with poor prognosis. Hence, the exploration of new biomarkers is essential for the early diagnosis and treatment of CRC.

With the development of high-throughput sequencing technology, huge amounts of databases such as TCGA (the cancer genome atlas) and GEO (gene expression omnibus) have emerged [2,3]. It is beneficial for us to analyze the genes of interest by bioinformatics, providing novel potential biomarkers. Heat shock factor 1 (HSF1) is a master transcription factor for modulation of the heat-shock response and the proteotoxic stress response. HSF1 has a carcinogenic role in regulating proliferation, survival, invasion and metastasis of cancer cells [4,5]. However, the roles of HSF1 in colorectal cancer are poorly understood. The latest research showed that HSF1 could induce protective autophagy for oxaliplatin resistance [6]. Our previous study reported that HSF1 promoted colorectal carcinogenesis by stimulating glutamine metabolism [7]. New exploration for the roles and driving forces of HSF1 in CRC is indispensable for targeting this molecular.

This study was dedicated to analyze the clinical characteristics for HSF1 in CRC by bioinformatics. More importantly, we were the first study to integrally reveal the driving forces for HSF1 overexpression in colorectal cancer by bioinformatics and experiments, providing theoretical basis for targeting HSF1.

## Materials and methods

### Expression analysis

The UALCAN [8] and Oncomine database [9] were used to investigate the different expression of HSF1 in colorectal cancer and normal tissues. Concomitantly, HSF1 mRNA expression was also compared at different CRC stages using the UALCAN, TISIDB [10] and GEPIA2 database [11]. *P* value cutoff is 0.05.

### Prognosis analysis

The relationship between the HSF1 expression and the prognostic significance including overall survival (OS), disease-free survival (DFS) and disease-specific survival (DSS) in colorectal cancer was validated by dedicated prognostic database – PrognoScan [12]. Meanwhile, The Human Protein Atlas (HPA) database [13] (http://www.proteinatlas.org/) was used to further explore the prognostic value. *P* value cutoff is 0.05.

### Functional enrichment analysis

The top 50 genes related with HSF1 in colon cancer and rectal cancer were speculated separately by UALCAN database. HSF1-related genes were input into DAVID [14] database for Gene Ontology (GO) and Kyoto Encyclopedia of Genes and Genomes (KEGG) pathways analysis. GO analysis contains biological process (BP), molecular function (MF) and cellular component (CC). The related results were visualized in Bubble plots. *P* value cutoff is 0.05.

### Immune feature analysis

The relationship between the HSF1 expression and the immune cell infiltration was analyzed by TIMER2 database [15]. *P* value cutoff is 0.05. The correlation between the expression of HSF1 and immunomodulators including PD-L1 (CD274), CTLA4 and PD-L2 (PDCD1LG2) was analyzed by TIMER2 and StarBase [16] database. *P* value cutoff is 0.05.

### Mutations analysis

The somatic mutation ratio of HSF1 was elucidated using TIMER2 database and COSMIC database [17]. Then, the online tool-ACLBI (https://www.aclbi.com/) was used to analyze the somatic mutation in 536 colorectal cancer samples from the TCGA database and explore the mutation characteristics of the top 20 high frequency mutated genes as well as HSF1. The data of cBioPortal database [18] showed the mutation types of HSF1 in CRC. The correlation between HSF1 mRNA expression and copy number variation was analyzed by cBioPortal database in colorectal cancer. *P* value cutoff is 0.05.

### Driver gene and promoter methylation analysis

The correlation between HSF1 expression and driver gene was illustrated through the TCGA portal database [19]. MethPrimer [20] was used to evaluate the CpG Island in HSF1 promoter. Subsequently, the association between the HSF1 expression and DNA methylation status in colorectal cancer was evaluated using cBioPortal database. Ultimately, DiseaseMeth version 2.0 [21] and UALCAN were used to estimate methylation levels of HSF1 between the CRC and corresponding adjacent tissues. *P* < 0.05 was regarded as statistically significant.

### Cell, antibodies and reagents

SW480 and HCT116 was obtained from the American Type Culture Collection (ATCC). SW480 was cultured in L-15 medium (GENOM, GNM41300) supplemented with 10% bovine serum at 37°C without CO2. HCT116 was cultured in McCOY’s 5A medium (GENOM, GNM16600) supplemented with 10% bovine serum at 37°C with 5% CO2. The following antibodies were used for Western blot and CO-IP: HSF1 (ab52757, Abcam), β-actin (4970 L, CST), Flag (F1804, Sigma), β-catenin (8480S, CST), HuR (Abcam, ab200342). JQ1(S7110) and I-BET-762 (S7189) was purchased from Selleck.

### Human tissue samples

Our study was approved by The Institute Research Medical Ethics Committee of Affiliated Hospital of Weifang Medical University. All patients signed informed consent forms. The samples of CRC patients were obtained from the Affiliated Hospital of Weifang Medical University from 1 January 2021 to 30 June 2021. None of the patients received other treatment before surgery. The samples were obtained immediately after surgery and stored at −80°C.

### SiRNA transfection

For this, 1 × 10^5^ cells was plated in 6-well plates and were transfected with siRNA by lipofectamine RNAiMAX (Invitrogen, 13778150) according to the manufacturer’s instructions. The siRNA was purchased from Genepharma (Shanghai, China) and used to knock down the expression of genes.

NC-S: UUCUCCGAACGUGUCACGU, NC-AS: ACGUGACACGUUCGGAGAA; β-catenin-1#S: GGACACAGCAGCAAUUUGU, AS: ACAAAUUGCUGCUGUGUCC; β-catenin-2#S: GCAGUUGUAAACUUGAUUA, AS: UAAUCAAGUUUACAACUGC. HuR siRNAs were purchased from RIBOBIO (Gua ngzhou, China). BRD4-1#S: GCGUUUCCACGUUUGGUACCGUGGAAACGC; BRD4-2#S: AGCUGAACCUCCCUGAUUA, AS: UAAUCAGGGAGGUUCAGCU.

### Western blot

After siRNA transfection, the cells in the 6-well plates were lysed by RIPA buffer. Cell lysates were separated and transferred to PVDF membranes. The PVDF membranes were incubated with primary antibodies at 4°C overnight. Then, the membranes were incubated with the secondary antibody conjugated with HRP (Abcam, ab6721) at room temperature for 1 h. Finally, the membranes were visualized with chemiluminescence (Solarbio, PE0010).

### RNA isolation, reverse transcription and quantitative PCR

The RNA was extracted in Trizol (Invitrogen, 15596026). After RNA quantification, 2 µg RNA was performed with reverse transcription via High-Capacity cDNA Reverse Transcription Kit (Applied Biosystems, 4375222). The real-time quantitative PCR was conducted by using UltraSYBR Mixture (cwbiotech, CW0957M) to determine mRNA expression. The primers used were listed as follows:

Tubulin-F: GAAGCAGCAACCATGCGTGA, Tubulin-R: AAGGAATCATCTCCTCCCCCA; HSF1-F: ACGGAGTTCCAGCACCCA, HSF1-R: CGCCACAGAGCCTCATTCT; HSF1-CDS-site1-F: GACCAAGCTGTGGACCCTC, HSF1-CDS-site1-R: CACTTTCCGGAAGCCATACAT; HSF1-CDS-site2-F: CCCGGATTCAGGGAAGCAG, HSF1-CDS-site2-R: CTGTCAGCAGGGAGATGGTG; HSF1-3’UTR-F: CGTGTCCTGTGGTTTGGTTG, HSF1-3’UTR-R: CCTGTCTTGTCCGTCCATCC.

### Co-immunoprecipitation (CO-IP)

For this, 5 × 10^6^ cells in 10 cm dish were lysed by NP40 buffer (Solarbio, N8031) containing 1× cocktail (Roche, 05892970001). Five hundred micrograms of protein was incubated with 5 µg RIgG or HSF1 antibody for overnight with rotating at 4°C. Then, 50 µl agarose beads was added to the mixture for 3 h with rotating at 4°C. The mixture was subjected to Western blot for detection.

### RNA immunoprecipitation (RIP)

For this, 1 × 10^7^ cells were lysed using RNA-Binding Protein Immunoprecipitation Kit (Millipore, No. 17–700) according to the manufacturer’s instructions. The lysates were immunoprecipitated with 5 µg RIgG or HuR antibody for overnight with rotating at 4°C. Total RNA was extracted and was performed with reverse transcription and real-time quantitative PCR.

### Statistics

Student’s *t*-test was performed for statistical significance analysis. *P* value <0.05 was considered as statistically significant. *P < 0.05, **P < 0.01, ***P < 0.001 and ****P < 0.0001.

## Results

Our study integrally analyzed the roles and driving forces for HSF1 in CRC by bioinformatics and experiments. We explored the expression and prognostic characteristics of HSF1 in CRC and performed the functional enrichment analysis as well as immune feature analysis. Subsequently, we explored the driving forces for HSF1 abnormal expression in CRC such as genetic alterations, epigenetic alterations and translation efficiency.

### The expression and the prognostic characteristics of HSF1 in CRC

**Figure 1.**
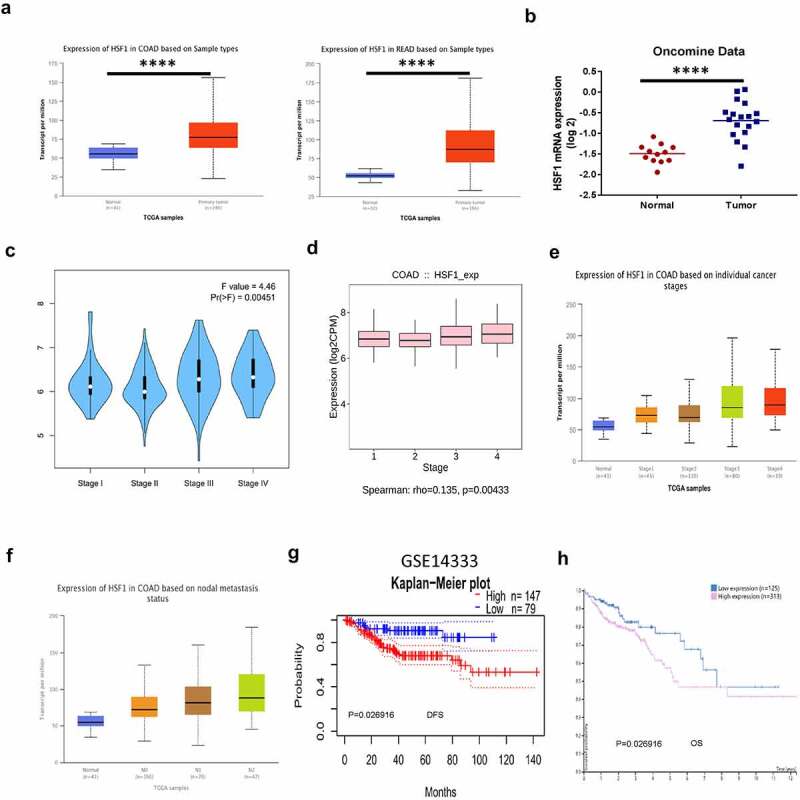
The expression and the prognostic characteristics of HSF1 in CRC. (a and b) The expression of HSF1 mRNA in CRC samples using UALCAN and Oncomine database. (c and d) The relative expression of HSF1 in COAD stages using GEPIA2 and TISIDB databases. (e and f) The relative expression of HSF1 in CRC stages and different lymph node metastasis using UALCAN database. (g) Kaplan–Meier curves demonstrated a negative correlation between HSF1 expression and disease-free survival (DFS) in CRC by PrognoScan database. (h) Kaplan–Meier curves demonstrated a negative correlation between HSF1 expression and overall survival (OS) in HPA database.

We first analyzed the expression of HSF1 mRNA in CRC by bioinformatics. HSF1 was significantly overexpressed in carcinoma than normal tissue by UALCAN and Oncomine database (Figure 1a-b). Moreover, HSF1 expression level was correlated with tumor stage and nodal metastasis status in colon adenocarcinoma (COAD) by GEPIA2, TISIDB, UALCAN database (Figure 1c-f). These results demonstrated that aberrant HSF1 expression may occur from early stages of COAD. Subsequently, we investigated the clinical significance of HSF1 expression in CRC patients. Based on the Kaplan–Meier survival curves, PrognoScan and HPA database analysis illustrated that high expression of HSF1 was significantly associated with poor disease-free survival (DFS) and overall survival (OS) in the CRC (Figure 1g and h). In summary, the aforementioned data indicated that HSF1 may be useful as a diagnostic and prognostic biomarker for CRC.

### Functional enrichment analysis

**Figure 2.**
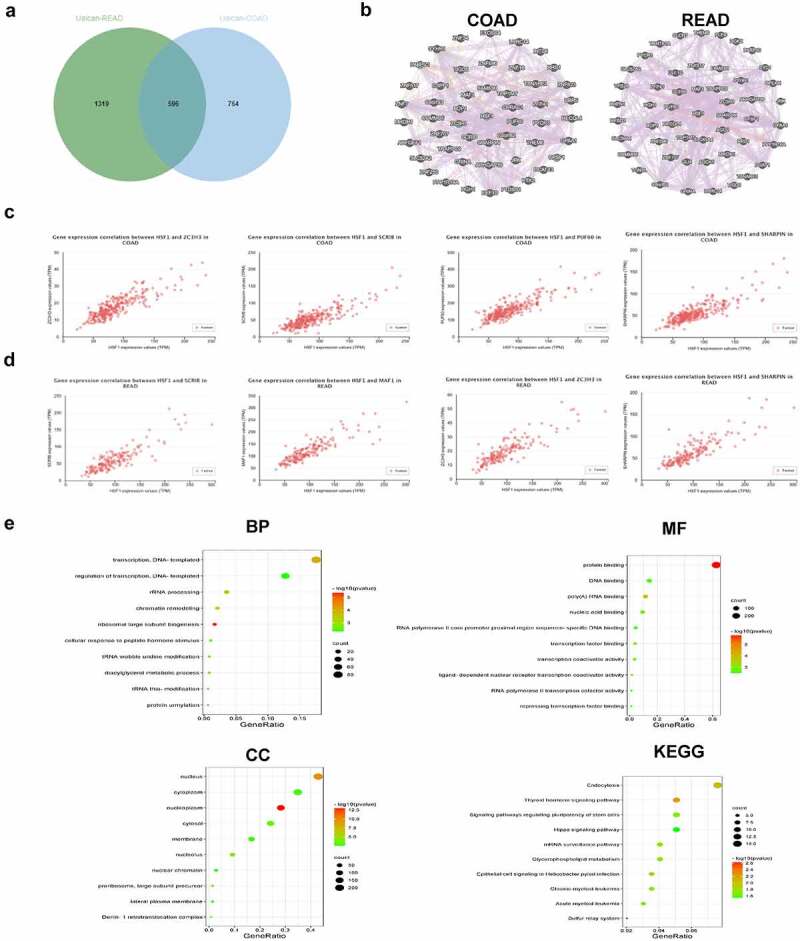
Functional enrichment analysis. (a) Venn diagram showed the intersection of HSF1-related genes in COAD and READ. (b) The regulatory networks between HSF1 and the top 50 related genes. (c and d) The correlation between HSF1 and the top four related genes in COAD (c) and READ (d) were analyzed using the UALCAN database. (e) GO and KEGG enrichment analysis about HSF1-related genes was shown based on DAVID database.

To further explore the roles of HSF1 in CRC, we screened out HSF1-related genes involved in COAD and rectum adenocarcinoma (READ) respectively using UALCAN database. We discovered 596 common members and visualized the top 50 gene networks through the GeneMANIA [22] database (Figure 2a and b). Among them, ZC3H3, SCRIB, PUF60 and SHARPIN showed the highest correlation with HSF1 expression in COAD (Figure 2c). While in READ, HSF1 expression was highly correlated with SCRIB, MAF1, ZC3H3 and SHARPIN (Figure 2d). Next, we performed the functional enrichment analysis of all relevant genes via the DAVID database. Figure 2e showed the top 10 most significantly enriched GO and KEGG pathways. In biological process (BP) term, transcription, DNA-templated and rRNA processing were mostly enriched. Nucleus, cytoplasm and nucleoplasm were significantly enriched in cellular components (CC). The molecular function (MF) mainly contained the protein binding, DNA and RNA binding. As for KEGG pathway, endocytosis, thyroid hormone and Hippo signaling pathway were significantly enriched.

### Immune feature analysis

**Figure 3.**
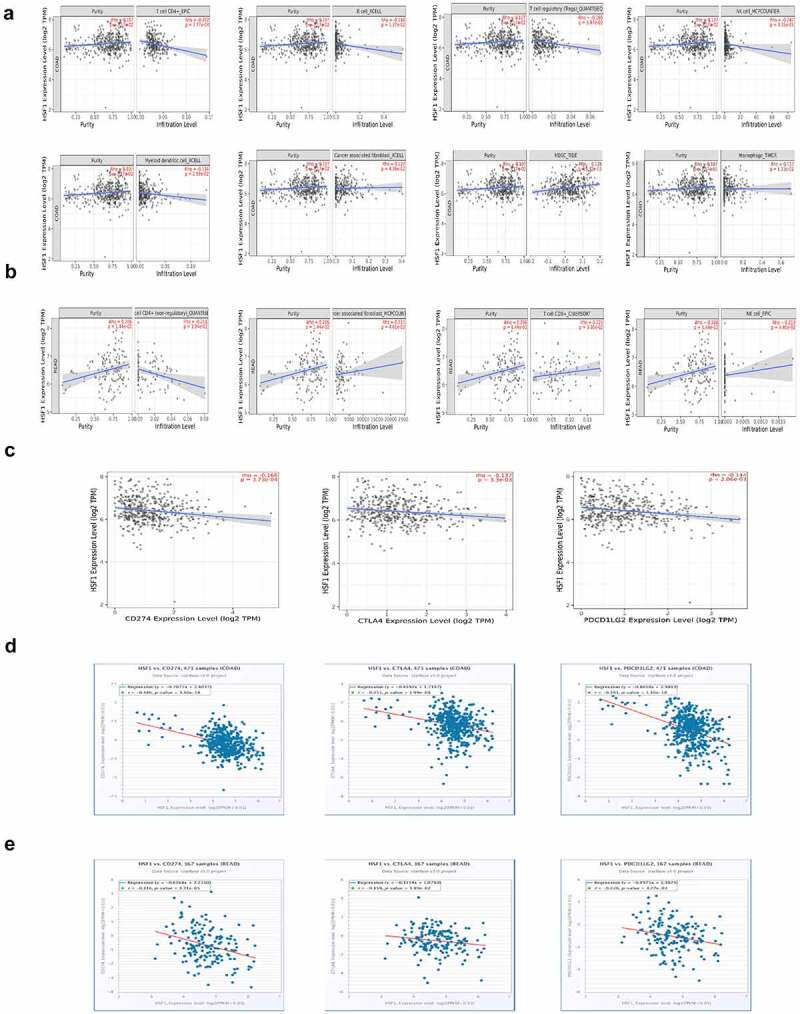
Immune feature analysis. (a) The correlation between HSF1 expression and immune cell infiltration was analyzed in COAD via TIMER2 database. (b) The correlation between HSF1 expression and immune cell infiltration was analyzed in READ via TIMER2 database. (c) The correlation between HSF1 expression and immunomodulators expression was analyzed in COAD via TIMER2 database. (d) The correlation between HSF1 expression and immunomodulators expression was analyzed in COAD via StarBase database. (e) The correlation between HSF1 expression and immunomodulators expression was analyzed in READ via StarBase database.

With the popularity of immunotherapy, we wondered whether HSF1 was correlated with immune features. First, we analyzed the infiltration level of immune cells via TIMER2 database. The results showed that HSF1 expression was negatively correlated with CD4 + T cell, B cell, Treg cell, NK cell and myeloid dendritic cell in COAD (Figure 3a). And HSF1 expression was positively correlated with cancer associated fibroblast (CAF), myeloid derived suppressor cells (MDSC) and macrophage in COAD. While in READ, HSF1 expression was negatively correlated with CD4 + T cell and positively correlated with cancer associated fibroblast (CAF), CD8 + T cell and NK cell (Figure 3b). More importantly, the expression of HSF1 was negatively correlated with the expression of immunomodulators such as PD-L1(CD274), CTLA4 and PD-L2(PDCD1LG2) (Figure 3c-e). Collectively, these results revealed that HSF1 may be involved in tumor immune regulation.

### Mutation status of HSF1 in CRC

Now that HSF1 has a critical role in CRC, we are keen to explore the driving forces for HSF1 to target it. In tumor cells, copy number variation (CNV) and mutation counts are important factors affecting the expression of related genes [23,24]. First, we illustrated the mutation features of HSF1 in tumors exploiting the TIMER2 database. The results demonstrated that the HSF1 mutation rate was low in all tumors including COAD-about 2.4% (Figure S1A). The top 20 high-frequency mutated genes in colorectal carcinoma were shown by online tool-ACLBI (Figure S1B) . In 536 colorectal cancer samples, the overall mutation rate was about 97.95% (525 out of 536) and the mutant frequency of APC was up to 75%, while the mutation rate of HSF1 was only 1%. The data of cBioPortal database showed the mutation types of HSF1 in CRC (Figure S2A). We summarized the CNV rate and gene expression rate of HSF1 in CRC through Catalog of Somatic Mutation in Cancer (COSMIC) database analysis. As shown in Figure S1C and S1D, the CNV rate of HSF1 was just 4.32%, while the gene expression rate was 29.67%. These data demonstrated that HSF1 mutation is one of the mechanisms leading to the upregulation of HSF1 expression among CRC patients. However, CNV does not fully explain the overexpression of HSF1 in CRC. Therefore, we need to further investigate other mechanisms that mediate HSF1 upregulation in CRC.

### The effect of genetic and epigenetic alterations on HSF1 overexpression

**Figure 4.**
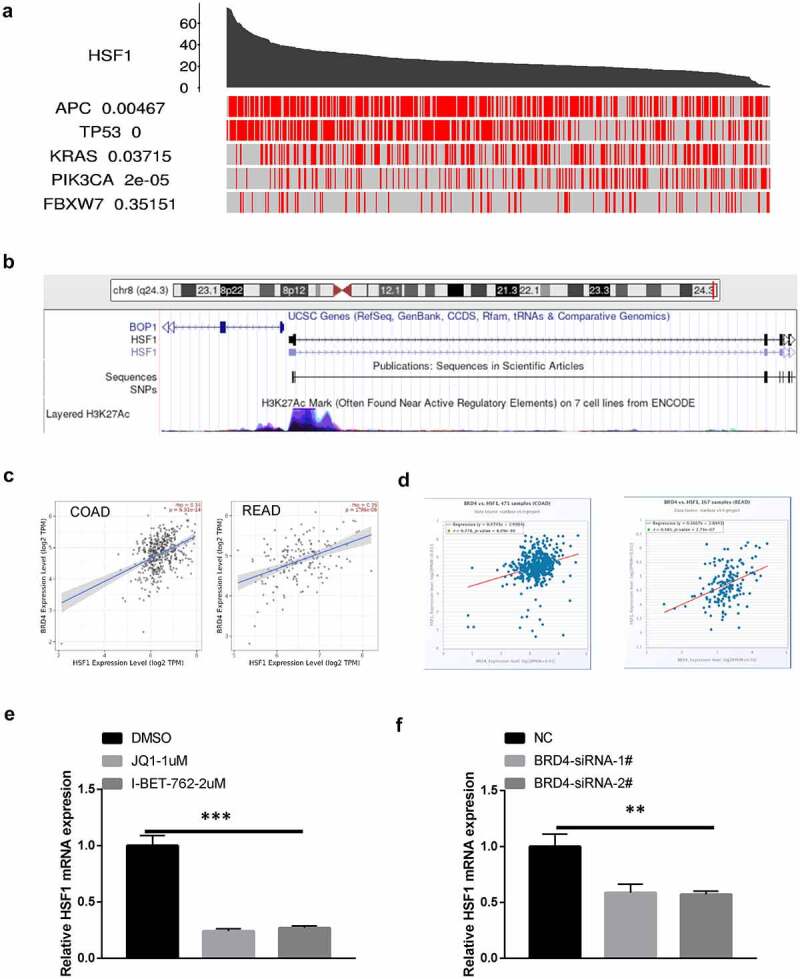
The effect of driver genes and super-enhancer on HSF1 expression. (a) The correlation between HSF1 expression and driver genes in COAD using the TCGA portal. (b) Analysis of H3K27ac signal in the transcription initiation site (TSS) of HSF1 by UCSC database. (c and d) The correlation between HSF1 and BRD4 expression in TIMER2 and Starbase database. (e) The effect of BRD4 inhibitor on HSF1 expression. (f) The effect of BRD4 knockdown on HSF1 expression.

To further investigate the reasons for the high expression of HSF1 in CRC, we analyzed the driver genes that are the critical nodes of regulatory networks and signaling pathways [25]. By TCGA portal database, we found HSF1 expression was correlated with multiple driver genes including *APC*, *TP53*, *KRAS* and *PIK3CA* (Figure 4a). Noteworthy, among them, *TP53* showed the highest correlation with HSF1 expression. Consistent with this result, HSF1 expression was significantly associated with mutant TP53 in the UALCAN database (Figure S2B). Besides genetic changes, tumor is also attributed to epigenetic alterations including histone modification, non-coding RNAs and DNA methylation. Histone modification such as acetylation, methylation, phosphorylation, etc. is critical epigenetic mechanism. In particular, high levels of histone H3 lysine 27 acetylation (H3K27ac) lead to the formation of super-enhancer, which is responsible for the control of key genes identity [26]. We observed that high H3K27ac signal exist in the transcription initiation site (TSS) of HSF1 by UCSC database (Figure 4b). Additionally, the expression of HSF1 is positively with the expression of BRD4, the master reader that binds to acetylated histones and regulates gene transcription (Figure 4c and d). Pharmacological inhibition or genetic deficiency of BRD4 could suppressed the expression of HSF1 (Figure 4e and f). All of these results implied the possible role of super-enhancer in regulating HSF1 expression.

**Figure 5.**
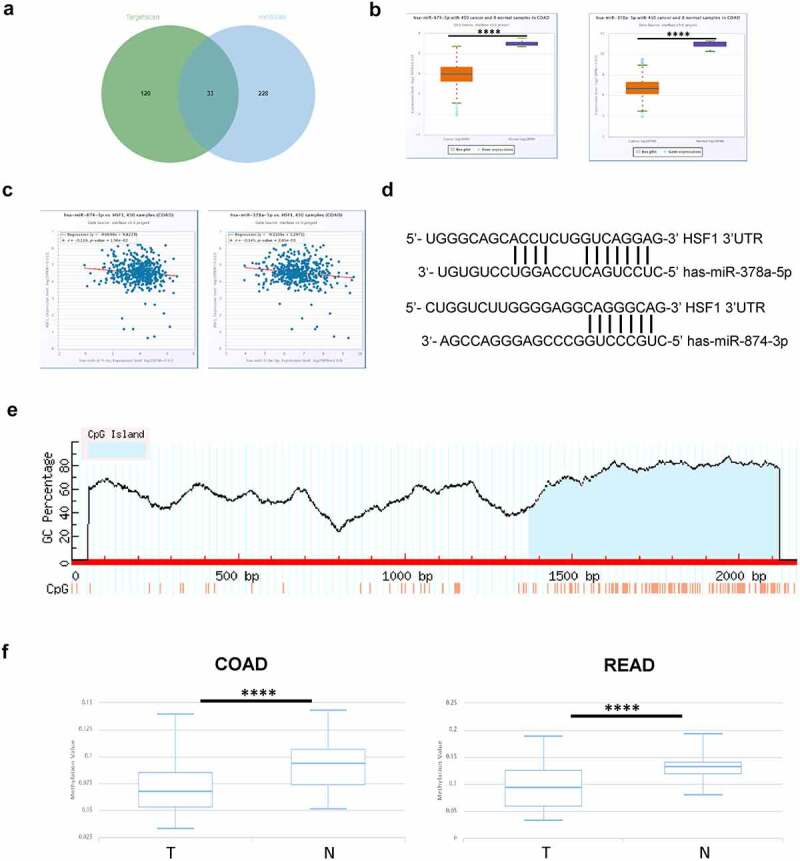
The effect of miRNA and DNA methylation on HSF1 expression. A. The potential miRNA for HSF1 by Targetscan and miRWalk database. (b) The expression of has-miR-378a-5p and has-miR-874-3p in COAD using StarBase database. (c) The correlation between the expression of has-miR-378a-5p and has-miR-874-3p and that of HSF1 in COAD using Starbase database. (d) Schematic representation of the predicted target site for miRNAs in HSF1 3’UTR. (e) A Schematic diagram of the CpG Island in HSF1 promoter region by MethPrimer website. (f) The methylation levels of HSF1 promoter in normal and CRC tumor groups by DiseaseMeth version 2.0.

Non-coding RNAs, especially microRNA (miRNA), could regulate gene expression by inducing mRNA degradation or inhibiting mRNA translation [27]. Subsequently, we screened out the potential miRNAs for HSF1 by TargetScan and miRWalk database [28,29]. There were about 33 common members (Figure 5a). Among them, only has-miR-378a-5p and has-miR-874-3p was downregulated and negatively correlated with the expression of HSF1 in CRC via StarBase database (Figure 5b-d). DNA methylation is closely related to gene expression [30]. Intriguingly, the MethPrimer website showed that there was a CpG Island in the HSF1 promoter region (Figure 5e). We further explored the relationship between HSF1 and DNA methylation in CRC by DiseaseMeth database. The analysis elucidated that HSF1 showed lower DNA methylation levels in CRC compared with normal tissues (figure 5f). A similar trend was observed in UALCAN database (Figure S2C). In addition, cBioPortal database revealed an obviously negative correlation between HSF1 expression and DNA methylation level in CRC (Figure S2D). Taken together, DNA methylation may provide a cause for HSF1 overexpression.

### HSF1 is regulated at the level of mRNA translation

**Figure 6.**
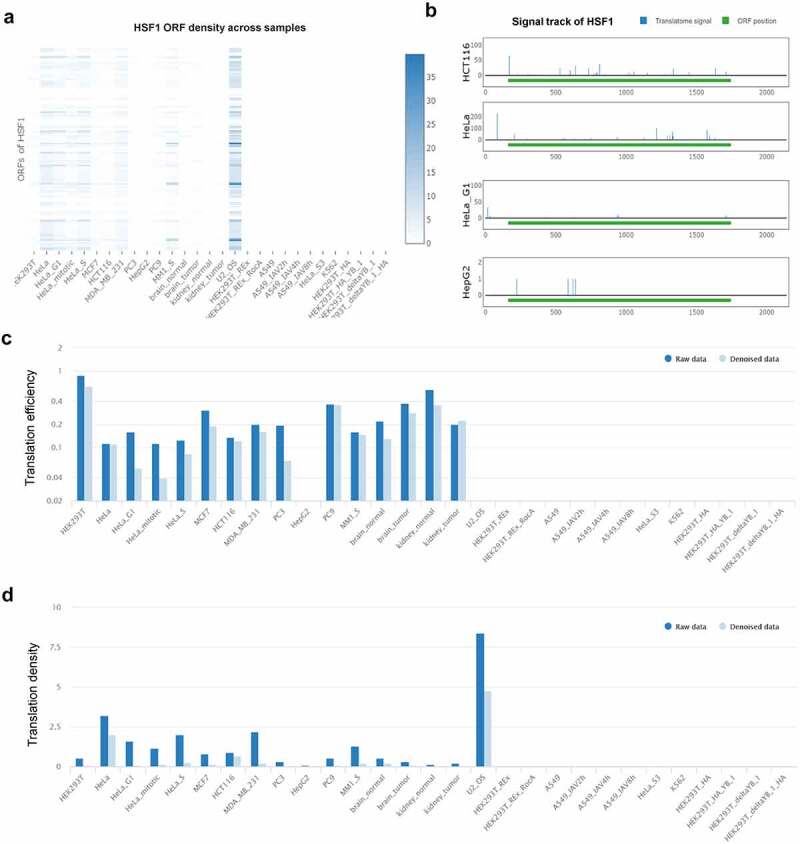
Analysis of the translation level of HSF1 mRNA in POSTAR3 database. (a) HSF1 ORF density across samples. (b) The translatome signal of HSF1 mRNA in different tumor cells. (c and d) The translation efficiency and density of HSF1 mRNA in different tumor cells.

Many key controls of gene expression occur at the level of mRNA translation ensuring the synthesis of cellular proteins under specific conditions such as heat shock and starvation [31]. We explored the translatome information of HSF1 mRNA by POSTAR3 database [32]. We found that there were multiple open reading frames (ORFs) for HSF1 mRNA among different tumor cells (Figure 6a). Next, we analyzed the most common transcript-ENST00000528838 and discovered that the translatome signal of this transcript in HCT116 was significantly increased (Figure 6b). Meanwhile, the translation efficiency and density of this transcript was higher in many tumor cells including HCT116 (Figure 6c and d). The above data demonstrated that HSF1 may be regulated at the level of mRNA translation in CRC.

### HuR co-expressed with HSF1 in CRC

**Figure 7.**
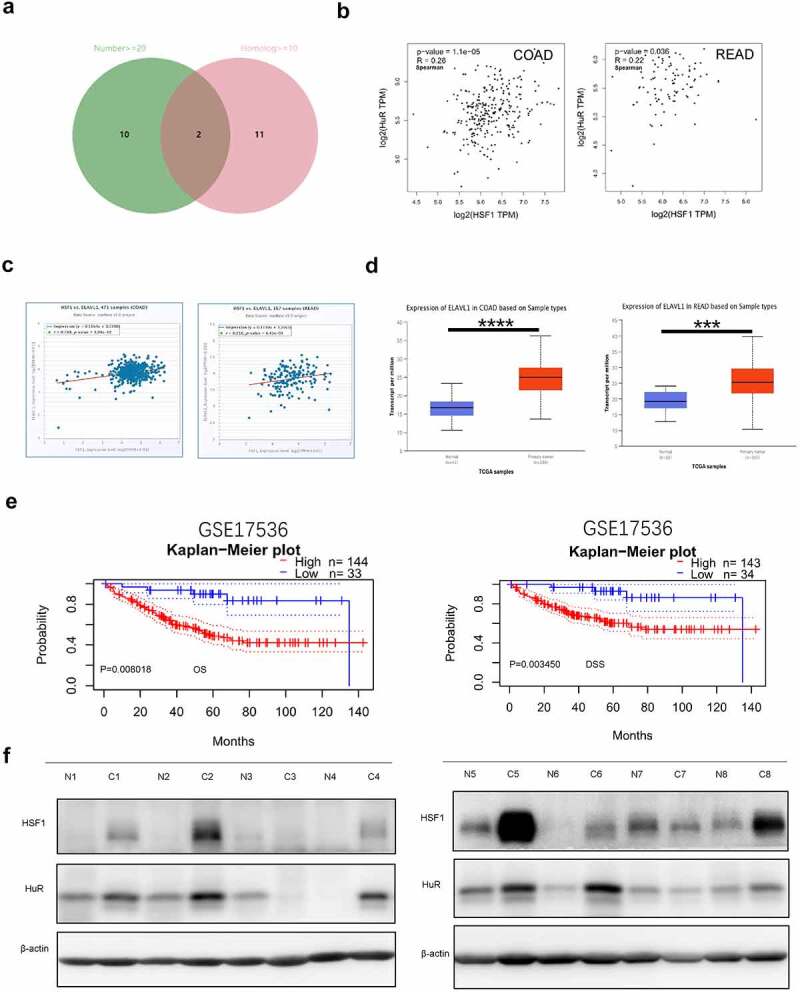
HuR co-expressed with HSF1 in CRC. (a) Venn diagram showed the RBP intersection in the RBPDB database (number of experiments (≥20) and human homologs (≥10)). (b and c) The correlation between ELAVL1 and HSF1 expression in CRC in GEPIA2 and StarBase database. (d) The UALCAN database showed the expression of ELAVL1 mRNA in CRC. (e) The correlation between ELAVL1 expression and CRC patient survival prognosis (overall survival (OS) and disease specific survival (DSS)) in the Prognoscan database. (f) The protein level of HSF1 and HuR in CRC tissues and corresponding normal tissues.

RNA binding proteins (RBPs) are commonly considered to combine RNA via globular RNA binding domains (RBDs) and alter the fate or function of the target RNA [33]. They are involved in many aspects of posttranscriptional regulation such as RNA processing, RNA transport, localization, degradation and translation efficiency [34]. Now that the translation level of HSF1 mRNA in colorectal cancer is high, we wanted to explore the certain RNA binding protein. To validate this hypothesis, we first screened out common RBPs using the RBPDB online database primarily focusing on collecting experimentally validated RBPs and RBDs [35]. The filter criteria were set to human, number of experiments (≥20) and Human Homologs (≥10). Finally, we selected 2 most common RBPs-ELAVL1 (HuR) and HNRNPA1 (Figure 7a). However, there is no significant correlation between the expression of HNRNPA1 and HSF1 in CRC (Figure S3A-B). While we found that the expression of HuR was positively correlated with that of HSF1 in CRC (Figure 7b-c). Furthermore, the expression levels of ELAVL1 were significantly higher in CRC than their corresponding normal tissues (Figure 7d and Figure S3C). Prognostic analyses from the PrognoScan database confirmed that patients with higher expression of ELAVL1 mRNA had significantly shorter overall survival (OS) and disease-specific survival (DSS) (Figure 7e). Subsequently, the relative protein expression of HSF1 and HuR in eight pairs of CRC tissues and their adjacent normal tissues were analyzed by Western blot assay. The results demonstrated that the expression of HSF1 and HuR was both significantly elevated and positively correlated in most of CRC patients (Figure 7f and Table 1). Table 2 shows the correlation between HSF1 expression and clinicopathological parameters in CRC patients (*n* = 8). Accordingly, HuR may be a potential RBP responsible for HSF1 overexpression.

**Table 1.** Correlation between HSF1 expression and HuR expression

	HSF1 (high)	HSF1 (low)
HuR (high)	6	0
HuR (low)	0	2
	*n*= 8	*P* < 0.05

**Table 2.** Correlation between HSF1 expression and clinicopathological parameters in CRC patients (*n* = 8)

Variables	HSF1 high (*n*)	HSF1 low (*n*)	*P* value
Age (years)			
>60	3	1	>0.9999
≤60	3	1	
Gender			
Male	4	2	>0.9999
Female	2	0	
Differentiation			
Well and moderate	5	2	>0.9999
Poor	1	0	
Lymph node metastasis			
N0-N1	5	2	>0.9999
N2-N3	1	0	
Infiltration depth			
T1-T2	3	0	0.4643
T3-T4	3	2	
Distant metastasis			
M0	6	2	>0.9999
M1	0	0

### HuR promoted the translation of HSF1 by binding to its CDS region

**Figure 8.**
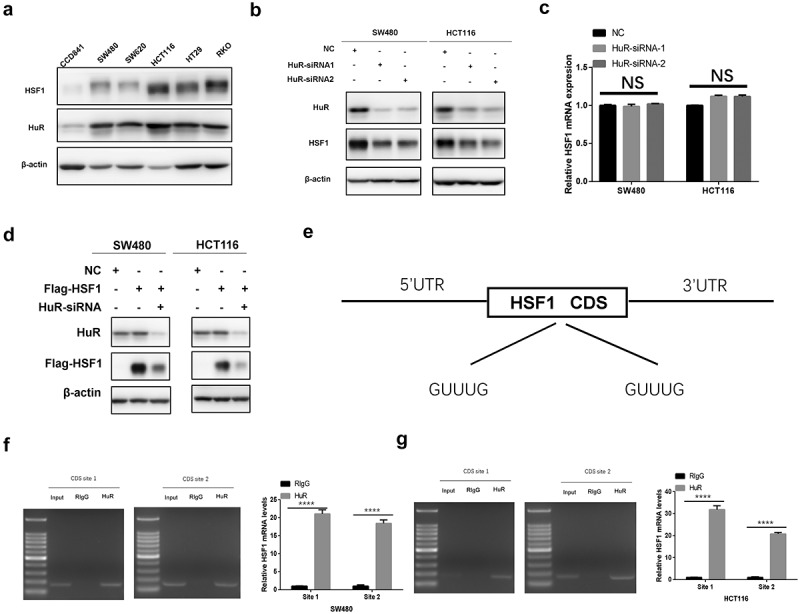
HuR promoted the translation of HSF1 by binding to its CDS region. (a) The protein level of HSF1 and HuR in CRC cell lines and normal colorectal epithelial cells (CCD841). (b) The protein level of HSF1 before and after HuR knockdown by Western blotting in CRC cells. (c) The effect of HuR knockdown on HSF1 mRNA expression. (d) The effect of HuR knockdown on exogenous HSF1 with CDS region. (e) Pattern diagram for two oligo (u) sequences in the CDS region of HSF1 mRNA. (f and g) RIP assay was conducted in HCT116 and SW480 cells using HuR antibody to verify the interaction between HuR and the oligo (U) sequences in the CDS region of HSF1 mRNA.

HuR is critical for regulating the translation process of oncogenic proteins [36]. To further verify whether HuR contributed to the translation of HSF1 mRNA, we first compared the protein expression of HSF1 and HuR in CRC cell lines. We found that both of them were upregulated in CRC cell lines compared with human normal intestinal epithelial cell CCD841 (Figure 8a). As expected, the protein level of HSF1 was downregulated after knockdown of HuR but not the mRNA level (Figure 8b-c). Normally, HuR interacted with one or several U or AU-rich elements (AREs) in the 3’-UTR (untranslated region) [37]. Interestingly, we observed that exogenous HSF1 with CDS region but not the 3’-UTR was also reduced after knockdown of HuR, demonstrating that HuR may promote the translation of HSF1 by binding to its CDS region (Figure 8d). Indeed, we found two fragments containing GUUUG sequences in the CDS region of HSF1 mRNA (Figure 8e). Moreover, we identified that HuR could directly bind to the two fragments of HSF1 CDS region as well as 3’-UTR by RNA immunoprecipitation (RIP) experiment (Figure 8f-g and Figure S4A). Collectively, these results implied that the translation of HSF1 was not only entirely dependent on the 3’-UTR but HuR could also play its role by the CDS region.

### β-Catenin co-operated with HuR to promote the translation of HSF1

**Figure 9.**
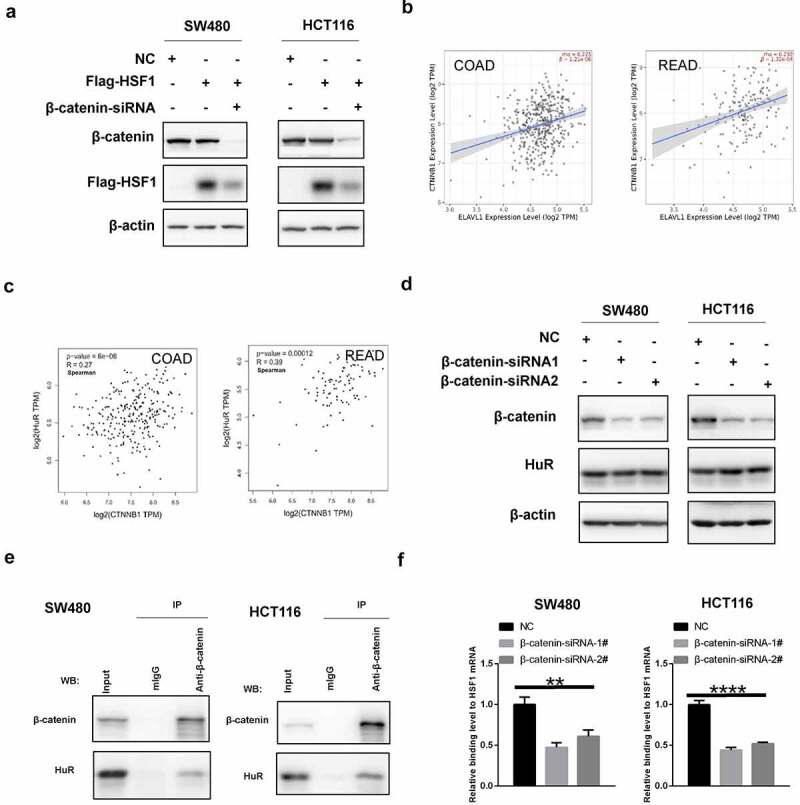
β-Catenin co-operated with HuR to promoted the translation of HSF1. (a) The effect of β-catenin knockdown on exogenous HSF1 with CDS region. (b and c) The correlation between β-catenin and HuR expression in CRC via TIMER2 and GEPIA2 database. (d) The effect of β-catenin knockdown on HuR protein expression. (e) The interaction between β-catenin and HuR via co-immunoprecipitation (CO-IP) experiment. (f) The binding levels of HuR to HSF1 mRNA by RIP assay before and after β-catenin knockdown.

Our previous study has reported that β-catenin (CTNNB1) can positively modulate HSF1 translation [38]. Therefore, we wonder whether there was a correlation between β-catenin and HuR. Similarly, exogenous HSF1 with CDS region but not the 3’-UTR was also reduced after knockdown of β-catenin (Figure 9a). Additionally, as shown in Figure 9b-c and Figure S4B, there was a positive correlation between CTNNB1 and HuR in CRC. Nevertheless, knockdown of β-catenin did not significantly alter the expression of HuR (Figure 9d). Notably, we discovered an interaction between β-catenin and HuR via co-immunoprecipitation (CO-IP) experiment (Figure 9e). Likewise, we found that the interaction between them scored highly by the HitPredict database [39] (Figure S4C). More importantly, the binding level between HuR and HSF1 mRNA was decreased after silencing β-catenin (figure 9f). Consequently, we hypothesized that HuR was able to stimulate the translation of HSF1 mRNA under the activity of β-catenin.

## Discussion

HSF1 is well known to be an important regulator of the heat shock reaction. The role of HSF1 in cancer has received widespread attention in recent years. Different from the heat shock response, HSF1 could drive a transcriptional program to endorse the malignant phenotype of cancer cells [4]. Our previous studies had verified that HSF1 is highly expressed in colorectal cancer patients. In present study, we further explore the roles and driving forces for HSF1 overexpression in CRC by bioinformatics and experiments.

We confirmed that HSF1 may be useful as a diagnostic and prognostic biomarker for CRC. Meanwhile, we performed the functional enrichment analysis, revealing the underlying molecular mechanism. Notably, our results implied that HSF1 was closely correlated with immune features in CRC. There was a discrepancy about the immune cell infiltration in COAD and READ. Extraordinarily, a significant positive correlation was both observed in COAD and READ between HSF1 expression and the abundance of cancer-associated fibroblast (CAF). Latest study reported that HSF1 is also essential for extracellular matrix remodeling in CRC by CAF [40], highlighting HSF1 vital roles in regulating tumor microenvironment. Interestingly, HSF1 could induce PD-L1 expression and enhance tumor growth in breast cancer [41]. However, our findings demonstrated that in CRC HSF1 was negatively correlated with the expression of immunomodulators such as PD-L1, CTLA4 and PD-L2. This result is consistent with previous findings that HSF1 expression was negatively correlated with tumor mutation burden (TMB) and microsatellite instability (MSI) in CRC [42]. Accordingly, HSF1 could be a predictive biomarker for immunotherapy in CRC, which is beneficial for selecting suitable patients to receive immunotherapy.

The present study about the driving forces for HSF1 overexpression in CRC is still limited. Tumorigenesis is a multistep process that involves genetic and epigenetic alterations, contributing to aberrant proteins and subsequent abnormal functions [43,44]. In this study, our data indicated that mutation of HSF1 may be partly responsible for the upregulation of HSF1 in CRC patients. While overexpressed HSF1 was subject to multiple driver genes in CRC, especially *TP53* which showed the highest correlation. This result is in accordance with Isermann’s study that mutp53 could unleash HSF1 function by eliminating the repressive WTp53-HSF1 axis [45]. Subsequently, we explored the effect of epigenetic alterations on HSF1 expression including histone modification, non-coding RNAs and DNA methylation. Unexpectedly, we found a super-enhancer in the TSS of HSF1 mRNA. Different from typical enhance, super-enhancer is a group of active-enhancer clusters which is more densely occupied by transcription factors and other histone regulators. Therefore, super-enhancer is indispensable for regulating the function of important genes and cell fate [46]. Targeting this super-enhancer may impose restrictions on the function of HSF1 in CRC. DNA methylation is one of the predominant epigenetic mechanisms affecting gene expression, which could regulate gene transcription levels depending on the methylation status of target genes [47,48]. Multiple databases showed a significant negative correlation between promoter methylation levels and HSF1 expression in CRC, providing an additional reason for HSF1 overexpression.

Protein expression is a complex process that is controlled by multiple steps, involving the transcription rate, mRNA degradation, translation regulation and protein degradation [49]. Bioinformatic analysis revealed a high translation efficiency of HSF1 mRNA in HCT116 cells. Our recent research has demonstrated that β-catenin inhibit miR-455-3p to increase m6A modification of HSF1 mRNA and facilitate its translation in CRC [38]. But there was still one problem unsolved that the expression of exogenous HSF1 without the 3’-UTR was also diminished after knockdown of β-catenin. Notably, in this study, we further found that this process was dependent on HuR. HuR binds transcripts primarily at U- or AU-rich RNA stretches which are usually located in the 3’-UTR of the target mRNA. Our results confirmed that besides 3′-UTR, HuR could also directly bind to the CDS region of HSF1 mRNA to promote its translation, providing new regulation mode for HuR. Previous studies showed that the effect of HuR was dependent on HSF1 expression [50]. These data supported a possibility that HSF1/HuR may constitute a feed-forward loop to coordinate their functions in CRC. Additionally, we identified a physical interaction between β-catenin and HuR, which has been reported previously [51,52]. More importantly, we revealed that knockdown of β-catenin attenuated the binding levels between HuR and HSF1 without influencing the expression of HuR, suggesting that HuR was able to stimulate the translation of HSF1 mRNA under the activity of β-catenin.

Collectively, our results shed light on the roles and driving forces for HSF1 overexpression in CRC. However, several issues still exist. First, the variation of some results could be due to differences in sample sizes, which is a limitation of online databases. Additionally, the specific role of β-catenin-HuR-HSF1 axis in CRC progression needs to be further confirmed by in vivo experiments.

## Conclusions

HSF1 may be useful as a diagnostic and prognostic biomarker for CRC. HSF1 was closely correlated with immune features. Genetic and epigenetic alterations contributed to HSF1 overexpression in CRC. More importantly, we demonstrated that HSF1 may be regulated at the level of mRNA translation by β-catenin-induced HuR activity.

## Supplementary Material

Supplemental Material

## Data Availability

All databases are freely available as public resources.

## Highlights

- HSF1 may serve as a diagnostic and prognostic biomarker for CRC.
- HSF1 was closely correlated with tumor immunity in CRC.
- HSF1 overexpression in CRC may be attributed to genetic and epigenetic alterations.
- HSF1 may be regulated at the level of mRNA translation by β-catenin-induced HuR activity.

## Abbreviations

ARE, AU-rich element; ATCC, American Type Culture Collection; CAF, cancer-associated fibroblast; CDS, coding sequence; CNV, copy number variation; COAD, colon adenocarcinoma; CO-IP, co-immunoprecipitation; COSMIC, Catalogue of Somatic Mutation in Cancer; CRC, colorectal cancer; DFS, disease-free survival; DSS, disease-specific survival; GEO, gene expression omnibus; GEPIA, gene expression profiling interactive analysis; H3K27ac, histone H3 lysine 27 acetylation; HPA, the human protein atlas; HSF1, heat shock factor 1; IARC, the international agency for research on cancer; KEGG, Kyoto Encyclopedia of Genes and Genomes; MDSC, myeloid-derived suppressor cells; MSI, microsatellite instability; ORF, open reading frame; OS, overall survival; PD-L1, programmed cell death 1 ligand 1; RBD, RNA binding domain; RBP, RNA binding protein; READ, rectum adenocarcinoma; RIP, RNA immunoprecipitation; TCGA, The Cancer Genome Atlas; TIMER, tumor immune evaluation resource; TMB, tumor mutation burden; TSS, transcription initiation site; UTR, untranslated region.
