# Supplementary figures and images for: Integrated bioinformatics and experiments reveal the roles and driving forces for HSF1 in colorectal cancer
Source: Bioengineered. 2022 Jan 10;13(2):2536–52. doi: 10.1080/21655979.2021.2018235 (PMC8974194; doi:10.1080/21655979.2021.2018235)

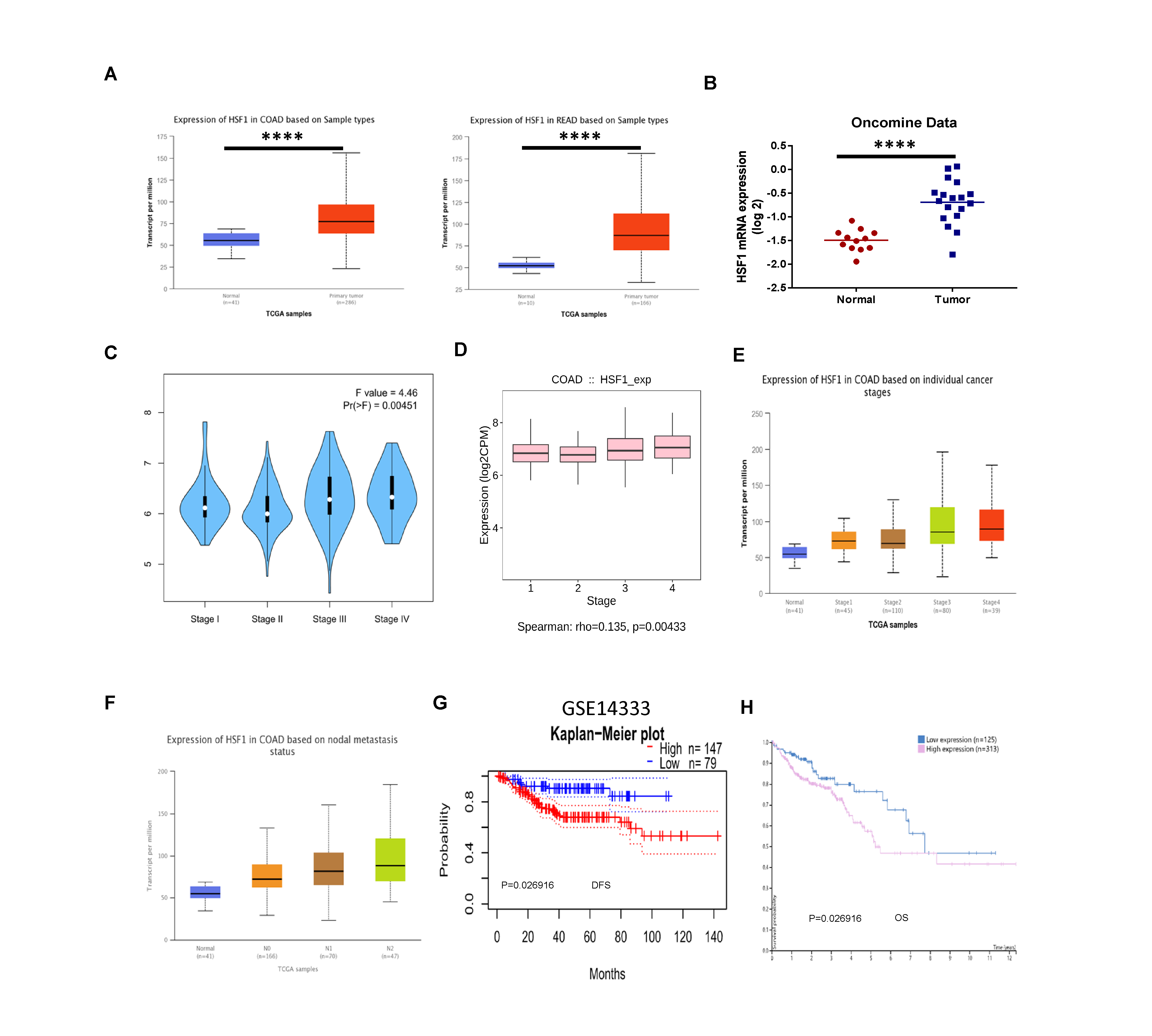

Supplement: Supplemental Material [file KBIE_A_2018235_SM4240.zip › supplementary/S-Figure 1.tif]

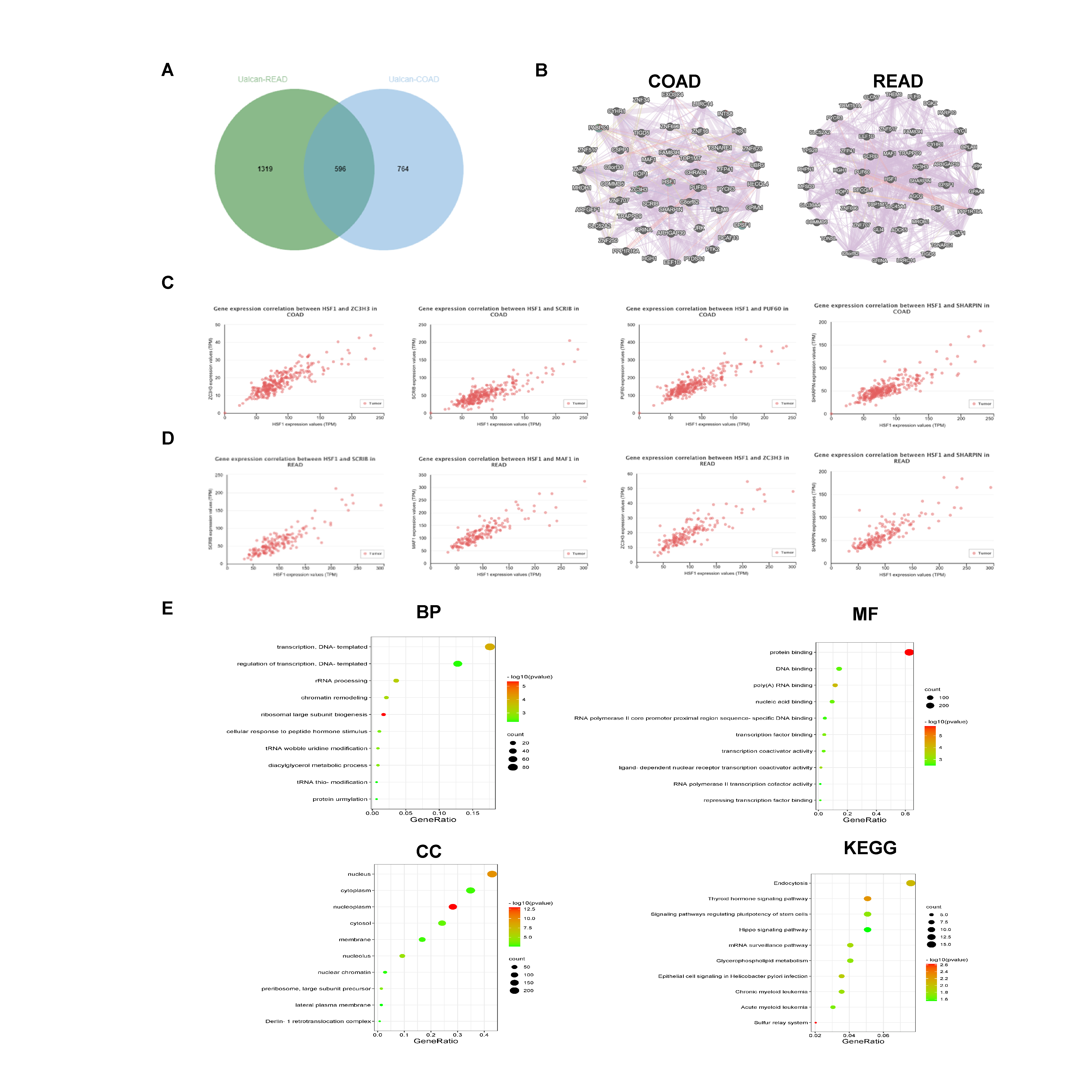

Supplement: Supplemental Material [file KBIE_A_2018235_SM4240.zip › supplementary/S-Figure 2.tif]

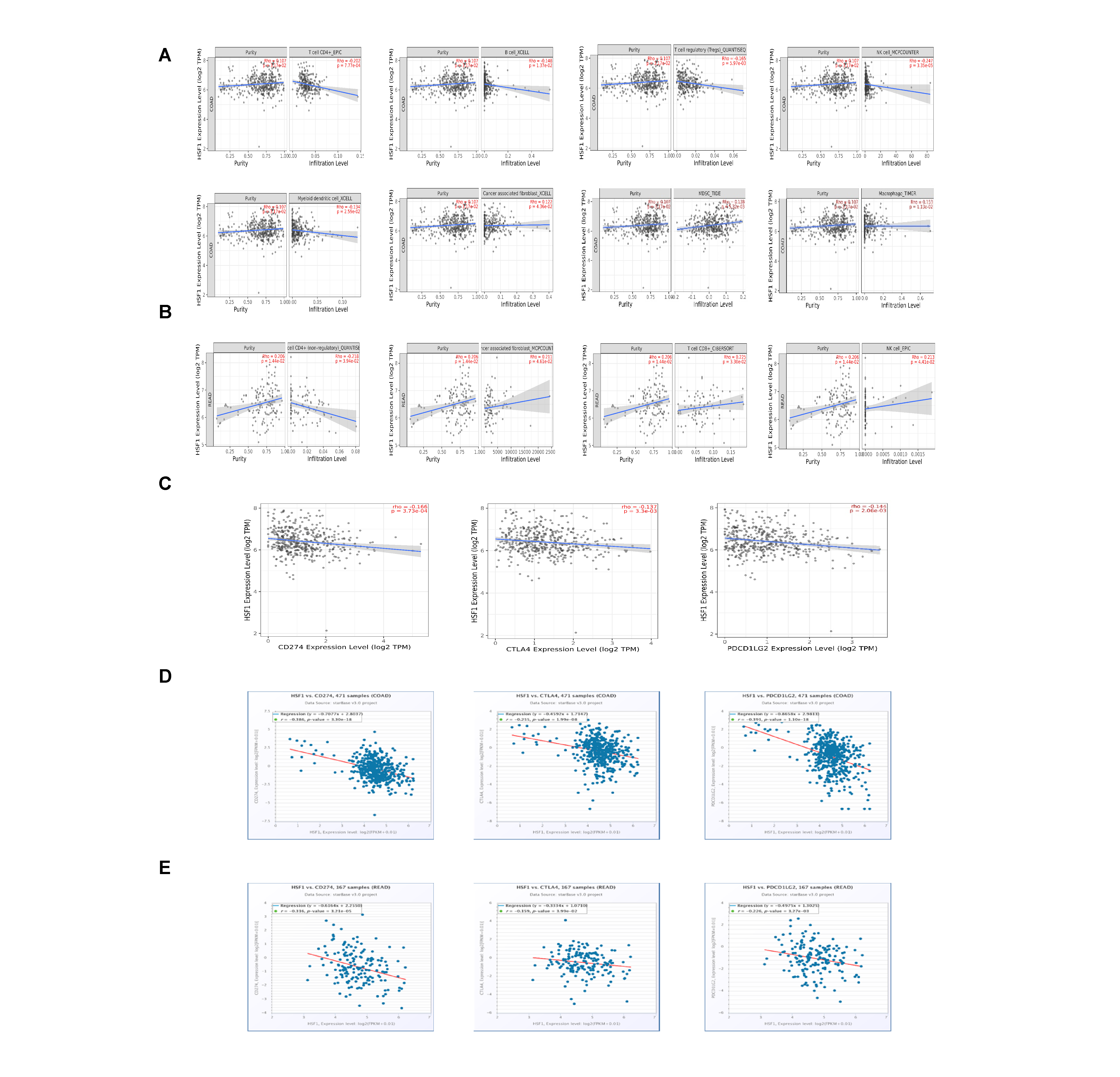

Supplement: Supplemental Material [file KBIE_A_2018235_SM4240.zip › supplementary/S-Figure 3.tif]

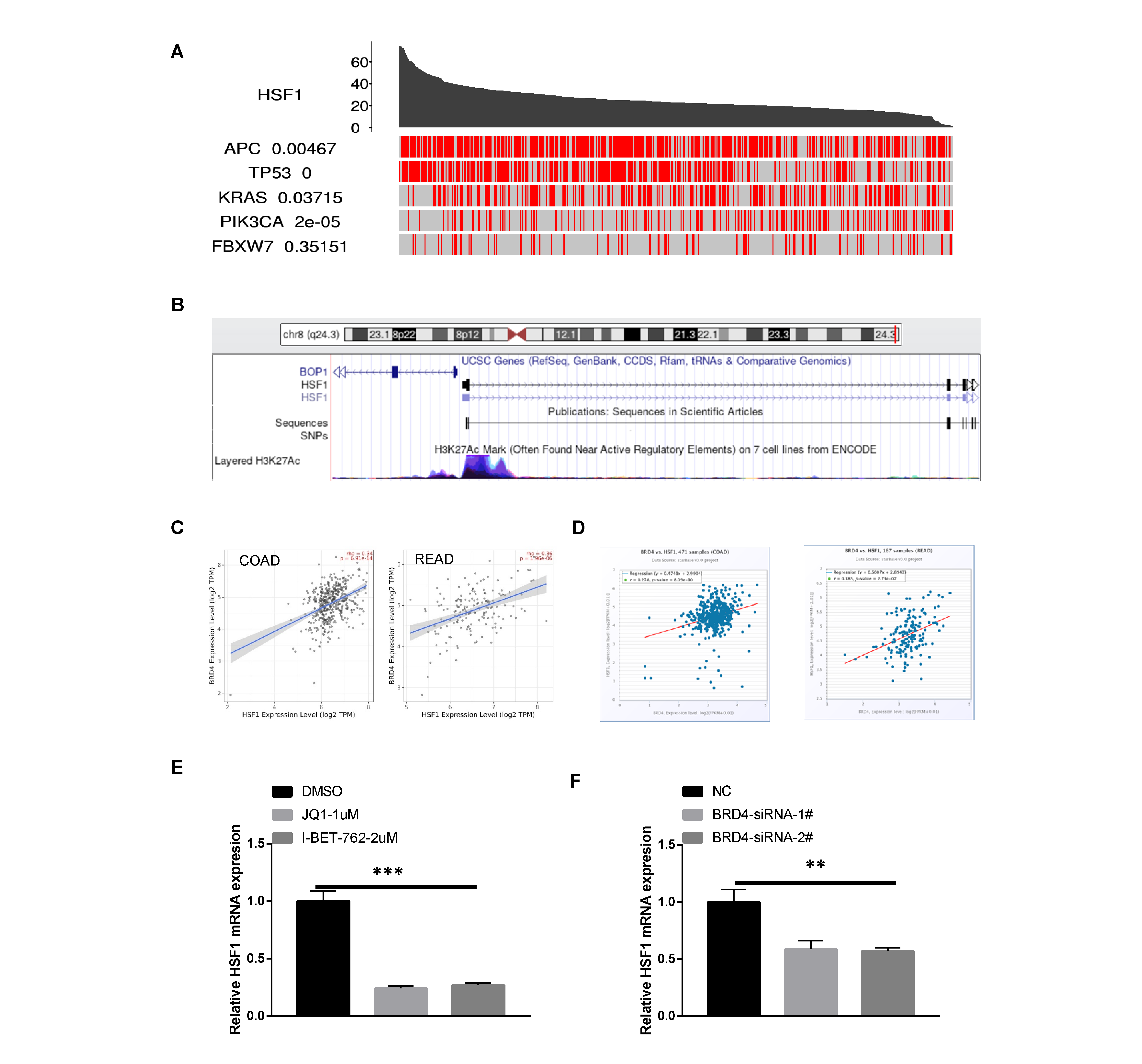

Supplement: Supplemental Material [file KBIE_A_2018235_SM4240.zip › supplementary/S-Figure 4.tif]
